# Higher levels of psychological distress are associated with a higher risk of incident diabetes during 18 year follow-up: results from the British household panel survey

**DOI:** 10.1186/1471-2458-12-1109

**Published:** 2012-12-23

**Authors:** Paula MC Mommersteeg, Raphael Herr, Wobbe P Zijlstra, Sven Schneider, François Pouwer

**Affiliations:** 1 CoRPS, Center of Research on Psychology in Somatic diseases, Department of Medical and Clinical Psychology, Tilburg University, Tilburg, The Netherlands; 2 MIPH, Mannheim Institute of Public Health, Social and Preventive Medicine, Medical Faculty Mannheim, Mannheim, Germany; 3 Department of Methodology and Statistics, Tilburg University, Tilburg, The Netherlands

**Keywords:** Type 2 diabetes, Psychological distress, Prospective, Risk factor, British household panel survey

## Abstract

**Background:**

Reviews have shown that depression is a risk factor for the development of type 2 diabetes. However, there is limited evidence for general psychological distress to be associated with incident diabetes. The aim of the present study was to test whether persons who report higher levels of psychological distress are at increased risk to develop type 2 diabetes during 18 years follow up, adjusted for confounders.

**Methods:**

A prospective analysis using data from 9,514 participants (41 years, SD=14; 44% men) of the British Household Panel Survey. The General Health Questionnaire 12 item version was used to assess general psychological distress, diabetes was measured by means of self-report. Cox proportional hazards regression models were used to calculate the multivariate-adjusted hazard ratio (HR) of incident diabetes during 18 years follow up, comparing participants with low versus high psychological distress at baseline (1991).

**Results:**

A total of 472 participants developed diabetes 18 year follow up. Those with a high level of psychological distress had a 33% higher hazard of developing diabetes (HR=1.33, 95% CI 1.10–1.61), relative to those with a low level of psychological distress, adjusted for age, sex, education level and household income. After further adjustment for differences in level of energy, health status, health problems and activity level, higher psychological distress was no longer associated with incident diabetes (HR=1.10, 95% CI 0.91-1.34).

**Conclusions:**

Higher levels of psychological distress are a risk factor for the development of diabetes during an 18 year follow up period. This association may be potentially mediated by low energy level and impaired health status.

## Background

Psychological distress has long been suspected as having important effects on the development of diabetes. The famous English physician Thomas Willis (1621–1675) for example, already noted that diabetes often appeared among persons who had experienced significant life events, sadness, or long sorrow [1]. In this context, psychological distress can be defined as “the unique discomforting, emotional state experienced by an individual in response to a specific stressor or demand that results in harm, either temporary or permanent, to the person” [2]. Psychological distress measures are sensitive screening instruments to detect mental disorders, affective disorders and anxiety disorders, in epidemiological studies and clinical populations [3,4]. In addition measurements of psychological distress reflect a general tendency toward expressing psychological distress rather than detecting psychological caseness. Thus screening for psychological distress goes beyond screening for either depression or anxiety and can have added value in examining general populations at risk.

In recent decades, most studies have focused on depression as a risk factor for type 2 diabetes. For example, meta-analyses by Knol et al. and Mezuk et al., showed that the risk for incident diabetes was 37-60% higher in depressed participants, compared to non-depressed controls [5,6]. Studies that have investigated different forms of distress associated with type 2 diabetes incidence point toward an increased risk for increased distress [7]. Concepts associated with psychological distress as stress [8], stress in daily life [9], Type A behavior [10], and anger temperament [11] show that mostly distressed men [9], but not women [8,10], or both men and women [11] were more likely to develop diabetes. In addition, whereas both psychological distress, as measured with the general health questionnaire (GHQ), and diabetes have been associated with increased mortality [12], the coexistence of both psychological distress and diabetes were associated with an increased mortality risk above and beyond of either factor alone [12].

Due to the various concepts of psychological distress used in previous studies, we sought to examine the association between psychological distress in general, using the GHQ12 and diabetes incidence. The aim of the present study was to examine the risk of psychological distress to develop type 2 diabetes, adjusted for potential confounders, using data from a large prospective and representative cohort study: the British Household Panel Survey [13]. We hypothesize that reporting increased psychological distress at baseline, is associated with an increased diabetes incidence, over the course of 18 years follow-up, independent of potential confounding variables.

## Methods

### Design and participants

Data are part of the British Household Panel Survey (BHPS), a nationally representative cohort of British households, recruited in 1991 and being re-interviewed each successive year (or *wave*) [13]. The main aim of the BHPS is to “further understanding of social and economic change at the individual and household level in Britain […], to identify, model and forecast such changes, their causes and consequences in relation to a range of socio-economic variables.” The British Household Panel Survey is conducted by the ESRC UK Longitudinal Studies Centre (ULSC), together with the the Institute for Social and Economic Research (ISER) at the University of Essex. In the present study, data collected between 1991 and 2009 were used (18 year follow-up cohort)[14]. The households were randomly selected from postcode districts in order to be nationally representative. In total 10,264 persons were annually interviewed starting in 1991, from age 16 and up. In each wave data were collected on several topics e.g. ‘income and wealth’, ‘housing’, and ‘health’ by a trained interviewer during a home visit. Data collection was done in accordance to the declaration of Helsinki, and the study was ethically approved by the University of Essex, Institute for Social and Economic Research [13]. For the present study, data on general psychological distress, diabetes presence, demographic characteristics, life style, and general health were extracted from the online database.

Of the 10,264 individuals who participated in 1991, respondents with missing data on the psychological distress questionnaire (*n* = 589/10,264; 5.7%) or who reported diabetes in 1991 (*n* = 179/10,264; 1.7%) were excluded. After exclusion of these two groups, the maximum number of participants available for analysis at baseline was 9,514 (Table 1). In total 45% of the non-diabetes group had information until the end of the study (Wave 18), and mean loss to follow-up time was 7.3 years. The percentage of dropouts was largest between wave 1 and 2 (10%), and between wave 2 and 3 (6%), and gradually decreased in consecutive waves, ranging between 5% and 2%. When comparing the characteristics of the dropout group between wave 1–17 to the group with information available in wave 18 (completers) at baseline, the dropout group showed no difference in psychological distress at baseline and was not different in BMI. Both groups comprised people with and without diabetes. However, the dropout group was significantly more likely to be older, male, not married, lower educated, have a lower average household income, nonwhite, less energetic (compared to age), have an impaired health status, were more likely to report health problems, more often reported an inactive/sedentary lifestyle, and were more likely to smoke. At the same time, the dropout group had a lower diabetes incidence of 4% (219/5225), compared to 6% (253/4289) in the completers group (*χ*^2^ = 14.6, *p*<.001). It must be noted that information on wave of diabetes presence and wave until last measurement was used in the analysis, thus optimizing the information available of people who dropped out during the 18 waves.

**Table 1:** Baseline characteristics of participants without diabetes, stratified by psychological distress

				**psychological distress**					
		**Total**		**High [4,021]**		**Low [5,493]**		**Test-value**^**1**^	***p*-value**
	**N**	**%/m**	***n/SD***	**%/m**	***n/SD***	**%/m**	***n/SD***		
*Sociodemographic factors*									
Age [years]	9,514	43.8	18.1	44.5	18.0	43.4	18.2	8.81	0.003
Sex [female]	9,514	54%	5,100	60%	2,417	49%	2,683	118.5	<0.001
Marital status [married]	9,502	59%	5,639	58%	2331	60%	3,308	5.00	0.025
Professional education	9,497							44.5	<0.001
Lower education		33%	3,101	36%	1,460	29%	1,641		
Medium education		33%	3,119	32%	1,267	34%	1,852		
Higher education		35%	3,277	32%	1,289	36%	1,998		
Race/ethnicity [non-white]	9,503	4%	352	4%	171	3%	181	5.94	0.015
Annual household income	9,514							73.9	<0.001
<15,000 £		44%	4,209	49%	1,979	41%	2,230		
15,000-<35,000 £		46%	4,358	42%	1,705	48%	2,653		
≥35,000 £		10%	947	8%	337	11%	610		
*Health*									
Energy	9,455							393.2	<0.001
More energetic		32%	2,987	25%	996	36%	1,991		
About the same		54%	5,124	53%	2,109	55%	3,015		
Less energetic		14%	1,344	22%	878	9%	446		
Health status	9,508	13%	1,200	19%	777	8%	423	284.1	<0.001
One/some health problems of which:	9,486	52%	4,960	59%	2,375	47%	2585	134.1	<0.001
Heart/blood pressure		11%	1,076	13%	538	10%	538	29.7	<0.001
Breathing problems, asthma, bronchitis		10%	963	13%	507	8%	456	47.3	<0.001
Skin conditions/allergies		11%	1,001	12%	488	9%	513	19.3	<0.001
Stomach/liver/kidneys		6%	524	7%	295	4%	229	44.71	<0.001
Problems with arms, legs, etc.		23%	2,138	27%	1,102	19%	1,036	97.2	<0.001
Difficulty in seeing		7%	656	9%	348	6%	308	33.54	<0.001
Difficulty in hearing		7%	698	8%	313	7%	385	2.04	0.153
Migraine or frequent headaches		8%	760	10%	414	6%	346	50.4	<0.001
Anxiety, depression, psych. problems	9,486	5%	473	10%	390	2%	83	329.3	<0.001
*Lifestyle*									
Leisure time activity ^2^	6,906							37.0	<0.001
Active		52%	3,609	50%	1,441	54%	2,168		
Moderately active		20%	1,391	19%	548	21%	843		
Inactive/sedentary		28%	1,906	31%	912	25%	994		
Smoking	9,506	30%	2,856	33%	1,331	28%	1,525	31.5	<0.001
BMI [kg/m^2^] ^2^	4,933	26.4	4.7	26.3	4.9	26.4	4.6	0.019	0.889
BMI categories ^2^	4,933								
Underweight < 18.5		1%	69	2%	31	1%	38	7.28	0.064
Normal weight 18.5 - 24.9		42%	2,077	44%	898	41%	1,179		
Overweight 25.0 - 29.9		38%	1,868	36%	731	39%	1,137		
Obese ≥ 30.0		19%	919	19%	385	19%	534		

^1^Test value Pearson *χ*^2^ for categorical variables and *F*-value for continuous scores.

^2^ Activity level was measured in wave 6, and BMI was measured in wave 13.

Baseline data were used for age, sex, marital status (married = 1, separated/divorced/widowed/never married = 0), smoking (yes=1), educational level (lower education, medium education (up to O-level) and higher education (A+ level), annual household income (<15,000 £, 15,000 -<35,000 £, and ≥35,000 £), health status, and health problems. Race/ethnicity was defined as: ‘white’ and ‘non-white’.

### Diabetes

In each consecutive wave, diabetes presence was assessed using a list of self-reported health problems (Do you have any of the health problems or disabilities listed on this card). In another study, self-reported diabetes was shown to reliably correlate with physician diagnosed diabetes [15]. There was no distinction between type 1 diabetes and type 2 diabetes, but as type 1 diabetes is generally diagnosed before the age of 25, most cases with type 1 diabetes are most likely excluded in the first wave.

### Psychological distress

General psychological distress was measured with the 12 item version of the General Health Questionnaire, the GHQ12 [4]. This questionnaire was self-completed by the participants during the home visit of the BHPS interviewer. The GHQ12 is used as a short screening instrument initially used to detect probable caseness of psychological disorders in epidemiological studies [4]. However, the GHQ reflects a general tendency toward expressing psychological distress rather than detecting psychological caseness, and it was used as an indicator of psychological distress in the present study. The reliability of the scale in the present sample was α = 0.85 (*N* = 9,675). The items are scored on a 1–4 item response scale, adapted to a dimensional scale, which coded 0-0-1-1 for the positive items, and 0-1-1-1 for the negative items (cGHQ12) [16]. In the present study a cut-off of ≤4 for the low-psychological distress group, and >4 for the high psychological distress group was used for the cGHQ12, based on previous large scale validation studies [4]. In the additional analysis, the continuous range of scores (0–12) were used.

### Measurement of potential confounders

Energy level, health status, health problems, and leisure-time activity were studied as potential confounders, as lower energy, poor health, and reduced physical activity can contribute to both higher levels of distress and a higher risk to develop diabetes [17].

### Energy

‘How energetic do you feel as compared to most people of your age?’ With three response categories: ‘more energetic’, ‘about the same’, ‘less energetic’.

### Health status

‘Does your health in any way limit your daily activities compared to most people of your age?’ (No=0/Yes=1).

### Health problems

Diabetes is often preceded by prodromal complaints, which may not be identified as being related to diabetes. Therefore different categories of self-reported health problems were recoded into ‘none=0’ versus ‘one/some=1’, based on the following categories: ‘heart/blood pressure’, ‘chest/breathing problems, asthma, bronchitis’, ‘skin conditions/allergies’, ‘stomach/liver/kidneys’, ‘problems with arms/legs etc’, ‘difficulty in seeing’, ‘difficulty in hearing’, and ‘migraine or frequent headaches’.

### Leisure-time activity

People reported in a ‘Leisure-time activities’ item how frequently they did leisure activities. A score of 2 (=Active) was assigned if someone reported ‘at least once a week’ to ‘play sport or go walking or swimming’, a score of 1 (=Moderately active) was assigned if a person reported ‘at least once a week’ to either ‘work in the garden’, ‘attend activity groups such as evening classes, keep fit, yoga etc.’, or ‘Do Do-It-Yourself, home maintenance or car repairs’. Finally a score of ‘0’ (=Inactive/sedentary) was assigned for the remaining answer categories.

### BMI and leisure-time activity from other waves

Data on body mass index (BMI) and leisure-time activity were not available in the first wave. Data on leisure-time activity was available from 1996 (wave 6), and BMI was available from 2004 (wave 13). Still, as both variables have been found to be related to diabetes development, these variables were used to predict diabetes incidence. There was a considerable number of missing cases in the waves of leisure-time activity (73% available, *n* = 6,906/9,514), and BMI (52% available, *n* = 4,933/9,514), and we choose to analyze BMI in a separate model in the additional analysis.

### Statistical analysis

Cox proportional hazards regression models were used to calculate the multivariate-adjusted hazard ratio’s (HR) of diabetes for the high-distress compared to the low-distress group using new diabetes cases in each consecutive wave [18]. Information on diabetes (present or absent), and time (coded as either first wave of diabetes presence, or last wave of diabetes absence) were used for the analysis. In total 12 cases were left-censored as there was missing information on diabetes in 1–3 waves preceding the the first wave of diabetes presence. As this time frame and number of cases were limited, these left-censored cases were not excluded.

We used two analysis strategies: first the effect of each covariate was examined separately, and second, the multivariate effect of a complete model was tested. In the first analyses, the individual HR of each covariate on diabetes incidence in the (age adjusted) model of high distress was investigated (Table 2, first columns, with 95% CI and *p*-value). The covariate adjusted HR of (age adjusted) high distress on diabetes incidence was reported (Table 2; column HR_covariate adjusted_). This was done by adding each covariate separately to the crude model. The change and the percent change in the log hazard ratio (B = Log HR) of high psychological distress (B_crude model_ – B_new model_) was calculated before and after adjustment for each individual potential confounder for the complete model available and reported in Table 2 as well (Table 2: Change (B_crude model_ – B_new model_) and % Change). To deal with loss to follow-up, a separate, additional, complete model was assessed for absolute or categorized BMI. A negative value depicts a *decrease* in LogHR for the high distress group after adjustment, whereas a positive score indicates an *increase* in the LogHR for the high-distress group after adjustment. We considered an absolute change in LogHR >1% to have a substantial effect as a confounder or mediator on the diabetes-associated risk of psychological distress, based on the present sample size, the number of covariates and adapted in line with the method used by Whooley and colleagues [19]. Second, a complete multivariate adjusted model was built. The covariates with a substantial effect (>1% absolute change in LogHR) were included hierarchically in three blocks of factors (sociodemographic, health, and lifestyle) (Table 3).

**Table 2:** Covariate adjusted hazard ratio table

**Covariate**	**HR**^**1**^	**95% CI**	***p*-value**	**HR**_**Covariate-adjusted**_^**2**^	**Change**^**3**^	**% Change**^**4**^
High distress [age adj.]	1.33	1.10-1.61	0.003	-	-	-
*Sociodemographic factors*						
Age	1.04	1.03-1.04	<0.001	1.33	-	-
Female sex [ref male]	0.74	0.62-0.90	0.002	1.37	0.030	10.36
Marital status [ref unmarried]	1.18	0.96-1.45	0.120	1.34	0.006	2.25
Lower education [ref medium]	1.29	1.02-1.64	0.037	1.29	−0.028	−9.37
Higher education [ref lower]	0.80	0.62-1.03	0.084			
Race/ethnicity [ref white]	2.63	1.27-4.02	<0.001	1.33	−0.003	−0.95
Annual household income <15,000 [ref 15,000 - <35,000]	1.10	0.89-1.35	0.398	1.31	−0.013	−4.52
Annual household income ≥ 35,000 [ref 15,000 - <35,000]	0.60	0.39-0.91	0.016			
*Health*						
More energetic [ref same energy]	0.74	0.59-0.93	0.009	1.14	−0.155	−54.25
Less energy [ref same energy]	2.09	1.65-2.66	<0.001			
Health status [ref ‘no’]	2.36	1.88-2.95	<0.001	1.19	−0.114	−39.90
One/some health problems [ref ‘none’]	1.70	1.38-2.10	<0.001	1.26	−0.055	−19.20
*Lifestyle*						
Inactive/sedentary [ref moderately active]	0.95	0.74-1.23	0.698	1.31	−0.013	−4.54
Active [ref moderately active]	0.72	0.57-0.89	0.003			
Smoking [ref no]	1.17	0.95-1.44	0.137	1.32	−0.006	−2.21
*Additional analysis*^5^						
BMI [kg/m^2^]	1.12	1.11-1.14	<0.001	1.29	0.013	5.38
BMI category ≤ 18.5 [ref 18.5-24.9]	2.19	0.95-5.06	0.065	1.29	0.009	3.61
BMI category 25.0-29.9 [ref 18.5-24.9]	2.09	1.55-2.81	<0.001			
BMI category ≥ 30 [ref 18.5-24.9]	6.13	4.61-8.14	<0.001			

^1^ The HR of each individual covariate is reported, adjusted for high distress and age. Health status and health problems are additionally adjusted for time dependent interactions (not shown).

^2^ The ‘HR_covariate adjusted’_ is the HR of high distress, adjusted for age and each covariate independently.

^3^ Change in the strength (logHR) of association of high distress before and after each covariate adjustment = Change in logHR = (B_crude model_ – B_new model_), and B = logHR.

^4^ The percentage change = 100*(Change in logHR)/B_crude model_. Absolute changes >1% were considered relevant, and included in subsequent multivariate regression models.

^5^ Information on BMI was not available until wave 13, and separate analysis were done for BMI, based on 452 diabetes events and 4780 cases. The age adjusted HR of high distress was 1.28 (95% CI = 1.03-1.57, *p* = .023) in that sample size.

**Table 3:** Covariate adjusted hazard ratios for high distress (top panel) and hazard ratio of the covariates for the complete model (lower panel) for 18-year follow-up diabetes incidence

	**HR**	**95% CI**	***p***
*Block 1: Sociodemographic factors*			
High distress	1.33	1.10-1.61	0.003
*Block 2: Health*			
High distress	1.10	0.90-1.34	0.342
*Block 3: Lifestyle*			
High distress	1.10	0.91-1.34	0.333
*Covariates [complete model]*			
*Sociodemographic factors*			
Age	1.03	1.02-1.04	<0.001
Female sex [ref male]	0.68	0.56-0.83	<0.001
Marital status	1.18	0.95-1.46	0.126
Lower education [ref medium]	1.21	0.95-1.54	0.115
Higher education [ref medium]	0.82	0.63-1.06	0.121
Annual hh income <15,000 [ref 15,000 - <35,000]	1.00	0.80-1.24	0.985
Annual hh income ≥ 35,000 [ref 15,000 - <35,000]	0.67	0.44-1.02	0.064
*Health*			
More energetic [ref same energy]	0.79	0.63-1.00	0.59
Less energetic [ref same energy]	1.54	1.18-2.02	0.002
Health status [ref ‘no’]	2.81	1.55-5.11	0.001
Health problems One/some [ref ‘none’]	1.43	0.77-2.63	0.255
*Lifestyle*			
Inactive/sedentary [ref moderately active]	1.06	0.82-1.38	0.655
Active [ref moderately active]	0.87	0.69-1.10	0.231
Smoking	1.01	0.82-1.25	0.915

Block 1: adjusted for age, female sex, marital status, education level, and annual household income.

Block 2: adjusted for block 1 + energy, health status, and health problems.

Block 3: adjusted for block 2 + activity in 1996, and smoking.

The proportional hazards assumptions of these models were verified using log-minus-log survival plots, Pearson correlations of the partial residuals, and the inclusion of interaction effects with process time. If the proportionality assumption was violated, nonproportional Cox models were used, by including interaction effects of the corresponding variable with process time, which corrects the violation of the proportionality assumption. This correction was used for health status and health problems. Additional analyses examined models with psychological distress as a continuous score, and models with interactions of sex or age with psychological distress. Statistical analyses were conducted using SPSS Statistics version 19.0 (IBM SPSS Inc., Chicago, IL, USA).

## Results

### Descriptives

Among the 9,514 included individuals, there were 472 incident cases of diabetes during the 18 year follow-up period. The incidence rate of new diabetes was 4.3 per 1000 person-years for this period of 18 years for the total sample. The incidence rate was 3.8 per 1000 person-years for low psychological distress and 4.9 per 1000 person-years for high psychological distress. In total 42% of the participants reported a high level of psychological distress at baseline (4,021/9,514). There was no difference in diabetes prevalence in 1991 between the high and low distress group (high psychological distress = 1.8%, low psychological distress = 1.5%, *χ*^2^ = 1.01, *p* = 0.313). The high psychological distress group reported to be less energetic compared to people of their age, have an overall poor health status and more health problems, including more psychiatric problems (Table 1). Moreover, the high distress group reported an inactive/sedentary lifestyle more often, had a higher prevalence of smoking, but showed no overall difference in average BMI, or BMI categories (Table 1).

### Diabetes incidence by psychological distress and covariate adjusted risk

**Figure 1:**
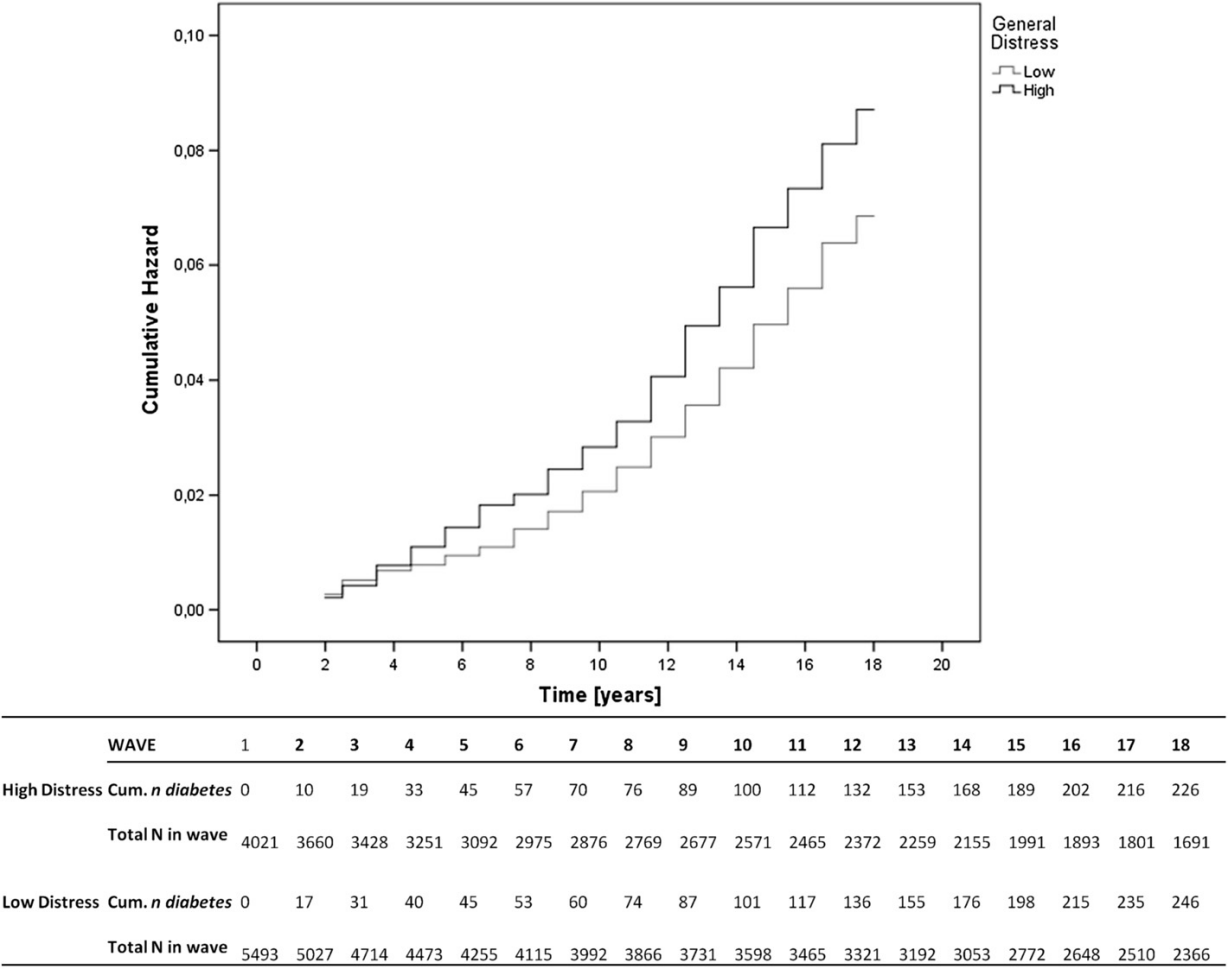
Cumulative hazard of diabetes stratified by general psychological distress.

There was a significantly increased incidence of diabetes in the high distress group (HR = 1.33, 95% CI = 1.10-1.61, *p*=0.003), adjusted for age (Figure 1). In Table 2, the hazard ratios of the covariates, adjusted for psychological distress and age (columns 1–3), and the covariate adjusted change in logHR for the high distress group are reported (column 4–6). After controlling for sex, marital status, and BMI, the adjusted LogHR for the high-distress group increased, whereas controlling for educational level, race/ethnicity, income, energy level, health status, health problems, leisure-time activity, and smoking lowered the LogHR for incident diabetes. Thus each of these factors explained some of the variance ascribed to the high distress group. The percentage change in LogHR is given for the effect of each covariate on the logHR of high psychological distress (Tables 1, 2), and a 1% absolute change was observed for sociodemographic factors: sex, marital status, education level and household income; health factors: energy, health status, and health problems, and lifestyle factors: leisure-time activity, smoking, and BMI.

### High distress and diabetes incidence

In Table 3 the adjusted model the hazard of the high psychological distress group on incidence diabetes, consecutively adjusted for each block of covariates is given. In the first step, the effect of high psychological distress was adjusted for four sociodemographic factors (age, sex, marital status, education level, and household income) which did not affect the risk (HR = 1.33, 95% CI = 1.10-1.61, *p*=0.003). In the second and third step, three health factors (energy level, health status, and health problems) and two lifestyle factor (leisure-time activity and smoking) were added to the model. The association between high distress and diabetes incidence in the final model became nonsignificant (HR = 1.10, 95% CI = 0.91-1.34, *p* =0.332). In the complete model younger age, female sex, having more energy or impaired health status were significant predictors of diabetes incidence (Table 3). Of the two time-dependent covariates impaired health status was significantly associated with a gradual decreased risk over time (HR = 0.95, 95% CI = 0.90-1.00, *p*=0.034), whereas no significant change over time for health problems was observed (HR = 1.00, 95%CI = 0.95-1.05, *p*=0.901) (data not shown in Table 3).

### Additional analyses

The *continuous* psychological distress score was entered in the additional analysis. Age adjusted HR showed an increased hazard for diabetes with each unit of increase in psychological distress (HR = 1.07, 95% CI = 1.03-1.10, *p*<0.001), which remained significant after controlling for sociodemographic factors sex, marital status, education level and income (HR = 1.07, 95% CI = 1.03-1.10, *p*<0.001), and became nonsignificant after controlling for health (energy, impaired health status, and health problems), and lifestyle (leisure-time activity, and smoking) (HR = 1.02, 95% CI = 0.98-1.05, *p*=0.309).

Since BMI was not introduced until wave 13, we decided not to include this variable in the main analysis. We performed an additional analysis, introducing the continuous BMI score to the complete adjusted model (not shown). Age adjusted high psychological distress was significantly associated with an increased diabetes incidence (HR=1.28, 95% CI = 1.04-1.58, *p* = 0.022), which was nonsignificant in the complete model (HR = 1.16, 95% CI = 0.93-1.44, *p* = 0.190). BMI was significantly associated with an increased hazard for diabetes (HR= 1.11, 95% CI = 1.10-1.13, *p*<0.001).

In two separate models potential interaction of age or sex with psychological distress was examined. There were no significant interaction effects of age and distress (HR_age*distress_= 0.99, 95% CI = 0.98-1.00, *p* = 0.193), and sex and distress (HR_sex*distress_= 1.32, 95% CI = 0.90-1.94, *p* = 0.152).

## Discussion

In this large prospective cohort study among men and women without known diabetes at baseline, a higher level of baseline symptoms of psychological distress, based on the GHQ 12 item version, were directly associated with a 33% increased hazard to develop diabetes during 18 year follow up. This association appeared to be confounded by energy level and health status after complete adjustment.

Earlier studies have examined the association between general psychological distress measures and incident diabetes, with varying results [7]. For example, both in the Copenhagen City Heart Study and two Japanese studies [8-10], particularly men with high levels of general distress but not distressed women were more likely to develop diabetes during follow up. In contrast, in the Whitehall II cohort, a high baseline GHQ score was not predictive of incident diabetes during a 10 year follow up period [20]. In the Whitehall II cohort study, a similar diabetes incidence rate was reported; 3.8 and 4.3 per 1000 person years in men and women respectively, compared to 4.3/1000 person years in the present study. The Whitehall II cohort was between 35–55 years at the start, whereas the BHPS included everyone > 16 years, with an average of 41 years. The Whitehall II cohort used odds ratios to predict the incidence of diabetes after an average of 10.5 years, whereas time dependent hazard ratios of 18 years were examined in the present study. Examining HRs of 18 waves is a more sensitive method of analysis, more likely to detect small differences.

The association between general psychological distress and incident diabetes in the present study was confounded by level of energy and impaired health status. There was an decreased hazard for an active lifestyle based on leisure-time activities with diabetes risk, compared to a moderate leasure-time activities, but no longer in the complete adjusted model. This is in contrast to other studies which consistently show an association between activity level, depression and diabetes [17,21]. In the present study, activity level was operationalized by leisure-time activity instead of a metabolic equivalent of physical activity and leisure-time activity was not measured until wave 6, which could have affected the strength of association with the variables measured at baseline. At the same time a person’s ‘energy level compared to age’ and whether or not a person’s health status was impaired could be proxy measures of a person’s health status and ability to be active, therefore more variables were present to determine health and potential activity at baseline. The overlap between health status, energy, leisure-time activity and psychological distress with diabetes is consistent with previous findings. For example, the study of Shirom and colleagues showed that vigor, a mood state comprising emotional energy, was related to a reduced diabetes risk 20 years follow-up, independent of depressive symptoms or anxiety [22]. Leisure-time physical activity has been found to mediate the association between emotional wellbeing and diabetes presence [23]. Rod and colleagues showed that respondents who reported high levels of psychological distress had less adequate health behaviors, such as being physically inactive [8]. Integrating increased activity into daily practice has been shown to be beneficial for mood as well as improve disease indicators [24]. Still, other factors such as a general healthier lifestyle, including a healthier diet with less saturated fat, reduced salt intake and increased fiber, and interventions aimed to reduce bodyweight have been effective in preventing diabetes [25], and could have played a role in the present study, though were not further investigated. Since adjustment for confounders does not provide information on whether a covariate is a mediator or a moderator in the association between psychological distress and diabetes incidence, we cannot draw firm conclusions on mediators or moderators. Rather, we can hypothesize that energy level, and health status may act as mediating factors in explaining the association between general psychological distress and incident diabetes, which remains to be investigated.

The observed association could also be confounded by the general psychopathology of affected participants, as psychological traits have been previously linked to incident type 2 diabetes [5-7]. In our study self-reported psychiatric morbidity was investigated, which was more prevalent in the high psychological distress group, however small sample sizes prevented inclusion in the main analysis.

Given that psychological distress is associated with an increased diabetes incidence, explanatory pathways may be via increased chronic stress. Chronic stress can increase the risk of type 2 diabetes directly, for example by long term activation of psychoneuroendocrine pathways with the release of catecholamines, such as adrenaline and norepinephrine and glucocorticoids as cortisol. This generally results in an increased hepatic glucose output, decreased insulin secretion and sensitivity, central accumulation of body fat, hypertension, and an adverse lipid profile [26,27]. Indirect pathways can operate through lack of adherence to healthy lifestyle behaviors, such as a low level of physical activity, unhealthy eating behaviors (e.g. higher saturated fat and carbohydrate intake), and smoking. The health associated factors ‘energy level compared to age’, and ‘impaired health status’ appeared to be of influence in the present study.

We suggest that future studies include clinical assessment of diabetes using an oral glucose tolerance test in association with measures of psychological distress, energy, and health status, and to observe potential biological mechanisms to further explore the association between general distress and diabetes incidence. At the same time interventions specifically aimed to increase activity level could potentially lead to reduced depressive symptoms and improve diabetes outcomes are currently being investigated [28,29]. Whereas screening for depression in diabetes appears to be of limited effect in improving diabetes distress or HbA(1c) levels [30], improving regimen adherence by a structured self-monitoring of blood glucose lead to significantly greater reductions in distress, compared to an active control group [31]. Clinical implications of the present study could be that a broader range of psychological distress symptoms needs to be taken into account in general practice, not just depressive symptoms. Primary prevention and anamnesis should also cover social history and biopsychosocial aspects of the patient. Caregivers and treatment providers (e.g. general practitioners and diabetologists) should therefore also take the case history, the person’s general health status, and perceived level of energy into account. Interventions aimed to improve lifestyle behavior (e.g. applying a diabetes prevention protocol to ‘real-world’ settings) were effective in attaining weight loss, which is associated to a reduced diabetes risk [32]. This type of intervention could easily be expanded by adding techniques specifically addressing psychological distress, yet whether addressing psychological distress has additional value in diabetes prevention programmes needs to be determined with a randomised controlled trial.

Despite the longitudinal character of the present study and the use of 18 waves, we cannot infer conclusions regarding causality from these results, which is a limitation of the present study. At the same time, there was no information available on the exact date of diabetes diagnosis which might be a limitation, but since information about diabetes was available in 18 consecutive waves we used the wave of first positive assessment of diabetes instead of the date. The variables related to drop out were also related to diabetes incidence, however in the dropout group the incidence rate of diabetes was lower. Therefore, it is difficult speculate about the effect of dropouts on the results. As our study was non-randomized there might be residual confounding, despite our attempted to adjust for the most important confounders in the multivariate analyses. Moreover, diabetes was measured by means of self-report in our study. Though studies have reported a strong association between physician’s report of diabetes and the patients self-report [15], there is still a considerable number of patients with type 2 diabetes who are generally not aware of the fact that they have type 2 diabetes. This is because type 2 diabetes has a long asymptomatic pre-clinical phase, often with prodromal symptoms, which frequently goes undetected. Of people with Type 2 diabetes, the proportion who are undiagnosed ranges from 30% to 90% [33]. We did take into account self-reported health problems that might be associated with prodromal complaints, e.g. problems related to blood pressure, skin conditions, kidney, difficulty seeing, though this was not further specified towards diabetes specific complaints. The present dataset may be subject to bias, as the diabetes prevalence at the onset of the study was low (1.7% in 1991), compared to the National diabetes prevalence (2.8% in 1996) [34]. Still, the selection of participants took place based on a random draw of postal code area’s. At the same time, the diabetes incidence rate of 4.3/1000 person years was representative of national findings of 4.4/1000 person years in 2005 [34]. Finally, we need to acknowledge that the assessment of physical activity was merely focused on leisure-time activities, and may not have represented people with a high active lifestyle or who were frequent sporters. As a result, activities such as (heavy) labor activities or household work were not covered.

Strengths of our study include not only the population based approach and the relatively large sample size, but also the long follow-up period, the use of Cox-proportional hazards model, which is more adequate and more sensitive in comparison to a logistic regression analysis. The availability of data from wave to wave was optimized by examining either the first wave of diabetes presence or the last wave of absence of diabetes. The study addresses an innovative question with potential clinical implications.

## Conclusion

Results of the present study show that persons with elevated levels of psychological distress are at increased risk to develop type 2 diabetes, potentially affected by low energy level and health status. These findings warrant a detection of psychological complaints beyond depression, and further investigation of life-style related interventions which include a module on psychological distress.

## Competing interests

SvS was supported by a small grant from Roche Diagnostics Deutschland GmbH, Mannheim, Germany, for a study on gestational diabetes. All other authors: Nothing to declare.

## Authors' contributions

PM contributed to the statistical analyses, wrote the results section, reviewed/edited the manuscript, contributed to the discussion, revised the manuscript. RH retrieved the data, conducted the statistical analyses, drafted the manuscript, reviewed/edited the manuscript, contributed to the discussion. WZ contributed to the statistical analyses, reviewed/edited the manuscript. SvS reviewed/edited the manuscript, contributed to the data analyses, and discussion. FP wrote the introduction and first draft of the discussion, reviewed/edited the manuscript. All authors read and approved the final manuscript.

## Pre-publication history

The pre-publication history for this paper can be accessed here:

http://www.biomedcentral.com/1471-2458/12/1109/prepub

## References

[B1] Willis T Pharmaceutice rationalis sive diatriba de medicamentorum operationibus in humano corpore 1675 Oxford: E Theatro Sheldoniano

[B2] Ridner SH Psychological distress: concept analysis Journal of advanced nursing 2004 45 5 536 545 10.1046/j.1365-2648.2003.02938.x 15009358

[B3] Hahn D Reuter K Harter M Screening for affective and anxiety disorders in medical patients - comparison of HADS, GHQ-12 and Brief-PHQ Psychosoc Med 2006 3 9 PMC2736509 19742274

[B4] Goldberg DP Gater R Sartorius N Ustun TB Piccinelli M Gureje O Rutter C The validity of two versions of the GHQ in the WHO study of mental illness in general health care Psychol Med 1997 27 1 191 197 10.1017/S0033291796004242 9122299

[B5] Knol MJ Twisk JW Beekman AT Heine RJ Snoek FJ Pouwer F Depression as a risk factor for the onset of type 2 diabetes mellitus A meta-analysis. Diabetologia 2006 49 5 837 845 10.1007/s00125-006-0159-x 16520921

[B6] Mezuk B Eaton WW Albrecht S Golden SH Depression and type 2 diabetes over the lifespan: a meta-analysis Diabetes Care 2008 31 12 2383 2390 10.2337/dc08-0985 19033418 PMC2584200

[B7] Pouwer F Kupper N Adriaanse MC Does emotional stress cause type 2 diabetes mellitus? A review from the European Depression in Diabetes (EDID) Research Consortium Discov Med 2010 9 45 112 118 20193636

[B8] Rod NH Gronbaek M Schnohr P Prescott E Kristensen TS Perceived stress as a risk factor for changes in health behaviour and cardiac risk profile: a longitudinal study Journal of internal medicine 2009 266 5 467 475 10.1111/j.1365-2796.2009.02124.x 19570055

[B9] Toshihiro M Saito K Takikawa S Takebe N Onoda T Satoh J Psychosocial factors are independent risk factors for the development of Type 2 diabetes in Japanese workers with impaired fasting glucose and/or impaired glucose tolerance Diabetic medicine 2008 25 10 1211 1217 10.1111/j.1464-5491.2008.02566.x 19046200 PMC2701561

[B10] Kato M Noda M Inoue M Kadowaki T Tsugane S Psychological factors, coffee and risk of diabetes mellitus among middle-aged Japanese: a population-based prospective study in the JPHC study cohort Endocrine journal 2009 56 3 459 468 10.1507/endocrj.K09E-003 19270421

[B11] Golden SH Williams JE Ford DE Yeh HC Sanford CP Nieto FJ Brancati FL Anger temperament is modestly associated with the risk of type 2 diabetes mellitus: the Atheroslcerosis Risk in Communities Study Psychoneuroendocrinology 2006 31 3 325 332 10.1016/j.psyneuen.2005.08.008 16198499

[B12] Hamer M Stamatakis E Kivimaki M Pascal Kengne A Batty GD Psychological distress, glycated hemoglobin, and mortality in adults with and without diabetes Psychosomatic Medicine 2010 72 9 882 886 10.1097/PSY.0b013e3181f6696e 20884891

[B13] Brice J Buck N Prentice-Lane E British Household Panel Survey User Manual Volume A: Introduction, Technical Report and Appendices 2010 Colchester: University of Essex

[B14] Institute for Social and Economic Research, British Household Panel Survey In Waves 1–18, 1991–2009 [computer file] Volume 7th Edition 2010 University of Essex, Colchester, Essex: UK Data Archive [distributor]

[B15] Kriegsman DM Penninx BW van Eijk JT Boeke AJ Deeg DJ Self-reports and general practitioner information on the presence of chronic diseases in community dwelling elderly A study on the accuracy of patients' self-reports and on determinants of inaccuracy. Journal of clinical epidemiology 1996 49 12 1407 1417 10.1016/s0895-4356(96)00274-0 8970491

[B16] Hankins M The reliability of the twelve-item general health questionnaire (GHQ-12) under realistic assumptions BMC Public Health 2008 8 355 10.1186/1471-2458-8-355 18854015 PMC2572064

[B17] Lysy Z Da Costa D Dasgupta K The association of physical activity and depression in Type 2 diabetes Diabetic Medicine 2008 25 10 1133 1141 10.1111/j.1464-5491.2008.02545.x 19046190

[B18] Klein M Kleinbaum D Survival Analysis: A Self-Learning Text 2005 New York Springer-Verlag: 2 edition

[B19] Whooley MA de Jonge P Vittinghoff E Otte C Moos R Carney RM Ali S Dowray S Na B Feldman MD Schiller NB Browner WS Depressive symptoms, health behaviors, and risk of cardiovascular events in patients with coronary heart disease JAMA 2008 300 20 2379 2388 10.1001/jama.2008.711 19033588 PMC2677371

[B20] Kumari M Head J Marmot M Prospective study of social and other risk factors for incidence of type 2 diabetes in the Whitehall II study Archives of internal medicine 2004 164 17 1873 1880 10.1001/archinte.164.17.1873 15451762

[B21] Koopmans B Pouwer F de Bie RA van Rooij ES Leusink GL Pop VJ Depressive symptoms are associated with physical inactivity in patients with type 2 diabetes The DIAZOB Primary Care Diabetes study. Family practice 2009 26 3 171 173 10.1093/fampra/cmp016 19321598

[B22] Shirom A Toker S Jacobson O Balicer RD Feeling vigorous and the risks of all-cause mortality, ischemic heart disease, and diabetes: a 20-year follow-up of healthy employees Psychosomatic medicine 2010 72 8 727 733 10.1097/PSY.0b013e3181eeb643 20716713

[B23] Sawatzky R Liu-Ambrose T Miller WC Marra CA Physical activity as a mediator of the impact of chronic conditions on quality of life in older adults Health and quality of life outcomes 2007 5 68 10.1186/1477-7525-5-68 18093310 PMC2246116

[B24] Barr-Anderson DJ AuYoung M Whitt-Glover MC Glenn BA Yancey AK Integration of short bouts of physical activity into organizational routine a systematic review of the literature American journal of preventive medicine 2011 40 1 76 93 10.1016/j.amepre.2010.09.033 21146772

[B25] Orozco LJ Buchleitner AM Gimenez-Perez G Roque IFM Richter B Mauricio D Exercise or exercise and diet for preventing type 2 diabetes mellitus Cochrane database of systematic reviews 2008 3 CD003054 10.1002/14651858.CD003054.pub3 18646086

[B26] Champaneri S Wand GS Malhotra SS Casagrande SS Golden SH Biological basis of depression in adults with diabetes Current diabetes reports 2010 10 6 396 405 10.1007/s11892-010-0148-9 20878274

[B27] Dandona P Aljada A Chaudhuri A Mohanty P Garg R Metabolic syndrome: a comprehensive perspective based on interactions between obesity, diabetes, and inflammation Circulation 2005 111 11 1448 1454 10.1161/01.CIR.0000158483.13093.9D 15781756

[B28] van der Heijden MM Pouwer F Romeijnders AC Pop VJ Testing the effectiveness of a self-efficacy based exercise intervention for inactive people with type 2 diabetes mellitus: design of a controlled clinical trial BMC Public Health 2012 12 331 10.1186/1471-2458-12-331 22559322 PMC3390268

[B29] Yates T Davies MJ Henson J Troughton J Edwardson C Gray LJ Khunti K Walking away from type 2 diabetes: trial protocol of a cluster randomised controlled trial evaluating a structured education programme in those at high risk of developing type 2 diabetes BMC Fam Pract 2012 13 46 10.1186/1471-2296-13-46 22642610 PMC3444401

[B30] Pouwer F Tack CJ Geelhoed-Duijvestijn PH Bazelmans E Beekman AT Heine RJ Snoek FJ Limited effect of screening for depression with written feedback in outpatients with diabetes mellitus: a randomised controlled trial Diabetologia 2011 54 4 741 748 10.1007/s00125-010-2033-0 21221528 PMC3052512

[B31] Fisher L Polonsky W Parkin CG Jelsovsky Z Amstutz L Wagner RS The impact of blood glucose monitoring on depression and distress in insulin-naive patients with type 2 diabetes Current medical research and opinion 2011 27 Suppl 3 39 46 21916532 10.1185/03007995.2011.619176

[B32] Johnson M Jones R Freeman C Buckley Woods H Gillett M Goyder E Payne N Can diabetes prevention programmes be translated effectively into real-world settings and still deliver improved outcomes? A synthesis of evidence. Diabetic medicine 2012 10.1111/dme.12018 PMC3555428 22998334

[B33] Taskforce IDF g Global Guideline for Type 2 Diabetes: recommendations for standard, comprehensive, and minimal care Diabetic Medicine 2006 23 6 579 593 16759299 10.1111/j.1464-5491.2006.01918.x

[B34] Massó González EL Johansson S Wallander MA Rodriguez LA Trends in the prevalence and incidence of diabetes in the UK: 1996–2005 Journal of epidemiology and community health 2009 63 4 332 336 10.1136/jech.2008.080382 19240084

